# Adverse childhood experiences in patients with schizophrenia: related factors and clinical implications

**DOI:** 10.3389/fpsyt.2023.1247063

**Published:** 2023-08-28

**Authors:** Lei Zhang, Nan Zhao, Minghuan Zhu, Minyi Tang, Weiqing Liu, Wenjuan Hong

**Affiliations:** Shanghai Pudong New Area Mental Health Center, School of Medicine, Tongji University, Shanghai, China

**Keywords:** schizophrenia, adverse childhood experiences, psychotic symptoms, personality pathology, abuse, neglect

## Abstract

The relationship between adverse childhood experiences (ACEs) and the development of psychotic symptoms is not well understood. Therefore, this study aimed to investigate the frequency and distribution of ACEs among patients with schizophrenia and their potential correlation with symptomatology and personality pathology. We conducted a cross-sectional study involving 571 patients with schizophrenia in Shanghai, China. Symptomatology was assessed using the Positive and Negative Symptoms Scale (PANSS) and personality pathology was assessed using the Personality Diagnostic Questionnaire Fourth Edition Plus (PDQ-4+). ACEs were assessed using the Child Trauma Questionnaire-Short Form (CTQ-SF). ACEs were highly prevalent, with 80.8% of the patients with schizophrenia reporting at least one ACE. The three most common types of ACE were physical neglect (69.8%), emotional neglect (28.2%), and emotional abuse (22.9%). For specific ACE, emotional abuse was significantly associated with PD traits, whereas emotional and physical neglect types of ACE was significantly associated with negative symptoms. A higher level of physical abuse was more commonly reported by men, younger individuals, and those with a higher level of antisocial PD traits. Higher levels of physical neglect were associated with more severe negative symptoms. ACEs are commonly observed in patients with schizophrenia. Therefore, it is strongly recommended that this clinical population be provided with a comprehensive assessment and individualized intervention for those exposed to specific ACEs.

## Introduction

Adverse childhood experiences (ACEs) have been identified as significant risk factors for the development of various mental disorders, such as schizophrenia. Numerous reviews have indicated that ACEs increase the risk of schizophrenia and affect the severity and type of psychotic symptoms (1–3). ACEs encompass a range of adverse experiences, including physical, sexual, and emotional abuse as well as neglect by caregivers (4). Researchers have consistently found that patients with schizophrenia are more likely to report ACEs than controls (5–7). Mall et al. (7) investigated a large sample of patients with schizophrenia and matched controls from the Genomics of Schizophrenia in the South African Xhosa people study and found that the odds of schizophrenia were 2.44 times higher among those who reported ACEs than those who did not. These findings suggest a potential causal relationship between ACEs and the development of schizophrenia, highlighting the need for further investigation of the association between specific and cumulative ACEs and schizophrenia in various countries.

The underlying mechanisms by which a specific ACE may contribute to the development of psychotic symptoms are not yet fully understood. Several hypotheses (8) have been proposed to explain this association. The stress-vulnerability model posits that early life stress (9), such as ACEs, can lead to neurobiological changes and alterations in stress response systems. These changes may increase an individual’s vulnerability to developing schizophrenia later in life, particularly when combined with a genetic predisposition or other environmental factors (10). In addition, the social-cognitive model suggests that ACEs can influence the development of maladaptive cognitive processes, including impaired social cognition (11–13) and personality disorders (PDs) (14–16). Cognitive deficits and PDs have been observed in patients with schizophrenia and may serve as mediators between ACEs and the manifestation of psychotic symptoms. Furthermore, the social defeat hypothesis (17, 18) proposes that ACEs contribute to the development of social defeat experiences that are associated with an increased risk of schizophrenia.

It is possible that PD traits mediate the relationship between ACEs and schizophrenia (19). ACEs may contribute to the development of PD traits, which, in turn, increase the risk or severity of schizophrenia symptoms. PD traits may shape an individual’s coping mechanisms, cognitive and emotional processing, and interpersonal functioning, which can impact the manifestation and course of schizophrenia. However, it is important to note that the relationship between ACEs, PD traits, and schizophrenia is complex and multifaceted. Not all individuals with ACEs develop PD traits, and not all individuals with PD traits develop schizophrenia. There are likely multiple pathways and factors involved, and current study is needed to better understand these relationships and potential mediating mechanisms.

Based on the existing literature, we hypothesize that ACEs are more prevalent among individuals with schizophrenia, and that different types of ACEs correspond to specific symptom profiles. This study aimed to explore the relationship between ACEs and schizophrenia with a specific focus on the frequency of ACEs in a clinical sample in China. In addition, by examining the existing literature and empirical evidence, this study aimed to shed light on the complex association between specific and cumulative ACEs and psychotic symptoms, ultimately contributing to a better understanding of the origin of the disorder and targeted preventive interventions. The decision to investigate the interrelationships between ACEs, PDs, and schizophrenia stems from the recognition of their potential interconnectedness. Prior research suggests that ACEs may play a significant role in the development of both PDs and schizophrenia. By studying these factors together, we aim to gain a more comprehensive understanding of how early-life experiences and personality characteristics may contribute to the onset and progression of schizophrenia. Such an integrated approach can offer valuable insights for developing more effective interventions and targeted support for individuals affected by these conditions.

## Methods

### Subjects

**Figure 1:**
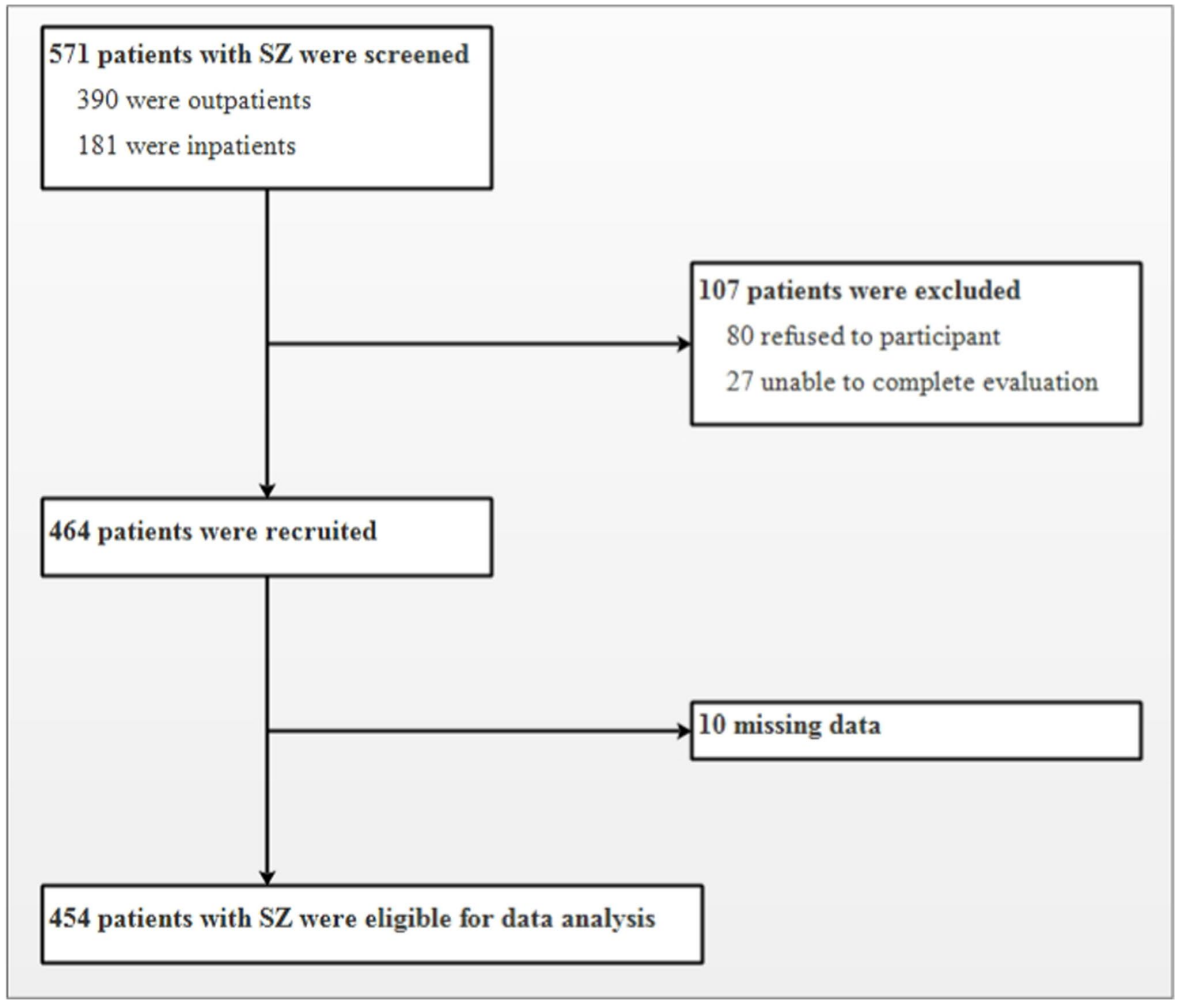
Enrollment of patients with schizophrenia (SZ).

A cross-sectional study of patients with schizophrenia was conducted at the Shanghai Pudong New Area Mental Health Center. Data were drawn from a consecutive clinical sample of adult patients to profile the frequency and association of ACEs with clinical symptoms and PDs. The study was conducted at a single site. Initially, 571 patients with schizophrenia were screened (280 men and 291 women). The inclusion criteria were as follows: (1) patients aged 18–60 years, (2) those able to understand the questionnaire used in this study, (3) willing to report childhood ACEs, and (4) under stable treatment conditions, defined as receiving consistent and appropriate antipsychotic medication for a minimum of 3 months prior to study enrollment, with no recent changes in medication dosage or treatment plan. The exclusion criteria were as follows: (1) severe or unstable physical disease, (2) current pregnancy, and (3) other situations considered by the investigators which make the patient ineligible. Patients who refused to participate in the study (46 cases refused to participate due to lack of time, 23 cases refused to participate due to concerns about information confidentiality, 10 cases refused to participate because these evaluations were not helpful to them, and 7 cases refused to participate due to poor self-reported status) or those without symptoms or signs were excluded (*n* = 107). After double entry and data verification, 454 patients were included in the analysis. A flowchart of the sample collection is shown in Figure 1.

The study was conducted in compliance with the Declaration of Helsinki and approved by the ethics committees of the Shanghai Pudong New Area Mental Health Center, Tongji University School of Medicine. To obtain consent, detailed oral and written information was provided to the patients and their informants to ensure that they fully understood the potential risks and benefits of the study. We confirm that we have read the journal’s guidelines on issues involved in ethical publication and affirm that this work is consistent with these guidelines.

### Measurement and instruments

Information on sociodemographic and basic clinical background was collected during the initial interview. Data on PD traits and ACEs were collected using self-administered questionnaires. Clinical psychotic symptoms were assessed using structured interviews.

ACEs were measured using the Chinese version of the Child Trauma Questionnaire Short Form (CTQ-SF) (15, 20, 21). The CTQ-SF comprises 28 self-report items that are assessed according to five childhood maltreatment subscales: emotional abuse, physical abuse, sexual abuse, emotional neglect, and physical neglect. The frequency with which each event occurred is rated on a 5-point scale ranging from 1 (never) to 5 (always), with higher scores indicating a higher level of ACE. The Chinese version of the CTQ-SF has been confirmed as a reliable and valid measure for assessing CTE in Chinese clinical samples (14, 15). Subjects who scored (i) 8 or above for physical abuse subscale, sexual abuse subscale, and physical neglect subscale; (ii) 10 or above for emotional abuse subscale; and/or (iii) 15 or above for emotional neglect subscale were considered as having experienced specific ACEs.

When implementing the CTQ-SF, it is essential to consider and control for potential recall biases. Recall biases refer to the distortion or inaccuracy of participants’ memories when reporting past experiences. Here are some strategies to minimize recall biases during the administration of the CTQ in this study. (1) Provide clear and specific instructions to participants about the timeframe they should consider when answering the questions. For example, specify that they should focus on their experiences during childhood or a specific age range. (2) Create a safe and supportive environment for participants to encourage honest and accurate responses. Assure confidentiality and emphasize the importance of their truthful and genuine recollection of their childhood experiences. (3) Encourage participants to indicate if they are uncertain or have difficulty recalling specific details rather than guessing or providing inaccurate information.

Clinical psychotic symptoms were measured using the Chinese version of Positive and Negative Symptoms Scale (PANSS) (22). The PANSS consists of 30 items divided into three subscales: positive psychopathology (PANSS-P; items P1–7), negative psychopathology (PANSS-N; N1–7), and general psychopathology (PANSS-G; G1–16). Each item (symptom) is rated on a 7-point Likert scale (1 = absent to 7 = extreme). Structured clinical interviews were conducted by three senior psychiatrists who received prior training required for this type of investigation. The inter-rater reliability for the PANSS ranged from 0.79 to 0.90 among the rating of the trained interviewers.

The PD traits were measured using the Personality Diagnostic Questionnaire-4+ (PDQ-4+) (23). The Chinese version of the PDQ-4+ has been shown to have high sensitivity (0.89) and moderate specificity (0.65) for screening patients with PD (24). The PDQ-4+ is a self-report questionnaire that assesses 12 specific PDs in the fourth version of the Diagnostic and Statistical Manual of Mental Disorders (DSM-IV). The PDQ-4+ consists of 107 true-false items and screens for 12 types of PDs (paranoid, schizoid, schizotypal, histrionic, narcissistic, borderline, antisocial, avoidant, dependent, obsessive, depressive, and negativistic PDs). Items are regarded as pathological responses and each item is rated one point. Higher subscale scores indicate a greater likelihood of having a certain PD trait.

### Statistical analyses

SPSS for Windows (version 20.0; IBM, Armonk, NY, United States) was used for data analysis. Statistical significance was set at *p* < 0.05. Quantitative variables are expressed as mean ± standard deviation (SD), and qualitative variables as frequencies (%). The mean scores of the neurocognitive tests in the three groups were converted to z-scores based on the mean and SD of the overall sample. The relationships among clinical symptoms, PD traits, and AECs were explored using Pearson’s correlation tests. A logistic regression model was fitted to identify factors associated with specific ACE. Variables in the logistic models were selected based on the age, sex, clinical symptoms of PANSS-P, PANSS-N and PANSS-G, 12 subtypes of PD traits and the association determined in the *χ*^2^ test. We reported the odds ratios (OR) according to 95% confidence intervals (CI) and *p*-values of Wald tests for the logistic models.

## Results

The sociodemographic and basic clinical information of the 454 participants is shown in Table 1. The participants were aged between 18 and 60 years, with mean age 26.8 ± 5.895 years. The proportion of men (44.5%) and women (55.5%) was generally equal. The average course of the illness was approximately 44 months, with 146 patients (32.2%) seeking their first visit during the present study. Table 2 lists the psychotic-related symptoms and personality disorder traits of the enrolled patients.

**Table 1:** Socio-demographic characteristics for patients with schizophrenia.

	Number/Means	%/SD
**Gender**		
Male	202	44.5%
Female	252	55.5%
**Age**		
Age (years)	26.80	5.895
**Education**		
Middle or high school	273	60.1%
College or higher	181	39.9%
**Marriage statues**		
Single, divorced, widowhood	360	79.6%
Married	94	20.7%
**Self-reported pre-illness characteristic**		
Introversion	255	56.2%
Middle type	142	31.3%
Extroversion	57	12.6%
**Family history of mental disorder**		
With family history	48	10.6%
Without family history	406	89.4%
**Visits and course**		
First visit	146	32.2%
2–5 visits	136	30.0%
6–10 visits	35	8.6%
>10 visits	133	29.3%
Course of illness (months)	44.59	51.516

**Table 2:** Clinical symptom and personality disorder traits for patients with schizophrenia.

	Means	SD
**Clinical symptoms (PANSS)**		
Positive symptoms	22.22	6.220
Negative symptoms	23.17	5.036
General symptoms	42.84	7.725
Total score	88.20	13.848
**Personality disorder traits (PDQ-4+)**		
Paranoid PD	3.29	1.708
Schizoid PD	2.69	1.406
Schizotypal PD	4.45	1.927
Antisocial PD	2.19	1.667
Borderline PD	4.76	2.254
Histrionic PD	3.83	1.895
Narcissistic PD	3.64	2.026
Avoidant PD	4.28	1.699
Dependent PD	3.80	1.843
Obsessive-compulsive PD	4.15	1.852
Passive-aggressive PD	3.31	1.639
Depressive PD	4.01	1.846

Table 3 shows that ACEs were highly prevalent in this study population, with 80.8% of patients with schizophrenia reporting at least one ACE. The three most common types of ACE were physical neglect (69.8%), emotional neglect (28.2%), and emotional abuse (22.9%).

**Table 3:** Adverse childhood experiences for patients with schizophrenia.

	Number/means	%/SD
**Scores**		
Physical abuse	7.81	3.065
Emotional abuse	6.37	2.339
Sexual abuse	6.11	2.124
Physical neglect	12.17	4.500
Emotional neglect	9.20	2.939
Total score	41.66	9.937
**Frequency**		
Physical abuse	86	18.9%
Emotional abuse	104	22.9%
Sexual abuse	75	16.5%
Physical neglect	317	69.8%
Emotional neglect	128	28.2%

All subjects who scored at or above 8 for physical abuse, sexual abuse, and physical neglect, 10 for emotional abuse, 15 for emotional neglect were considered as having experienced specific adverse childhood experiences.

**Figure 2:**
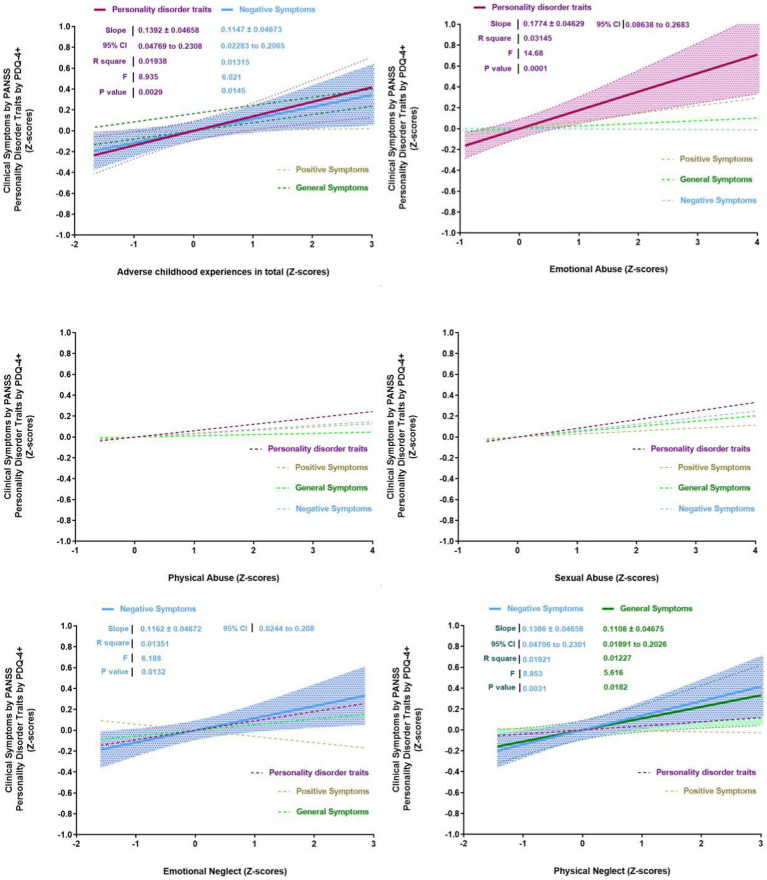
Correlations between personality disorder traits, clinical symptoms and adverse childhood experiences.

Overall, the total ACE scores were significantly associated with PD traits and negative symptoms on the PANSS subscale. For specific ACE, emotional abuse was significantly associated with PD traits, whereas emotional and physical neglect types of ACE was significantly associated with negative symptoms. In addition, physical neglect was significantly associated with the general symptoms (Figure 2).

Physical abuse was significantly associated with sex, age, and antisocial PD traits. Specifically, a higher level of physical abuse was more likely to be reported by men, younger individuals, and those with higher levels of antisocial PD traits (Table 4). Higher levels of physical neglect were associated with more severe negative symptoms.

**Table 4:** Associations between the specific adverse childhood experiences and demographic and clinical variables.

Variables	Analysis						
	Beta	S.E.	*β*(OR)	95%CI for *β*(OR)		*χ*^2^	*p*
**Physical abuse as the dependent variable**							
Age	−0.050	0.024	0.951	0.908	0.996	4.500	0.034
Sex (male)	0.624	0.253	1.866	1.136	3.063	6.079	0.014
Antisocial PD	0.182	0.099	1.200	0.988	1.456	3.393	0.065
**Emotional abuse as the dependent variable**							
Schizotypal PD	0.173	0.089	1.188	0.998	1.415	3.766	0.052
Antisocial PD	0.196	0.093	1.217	1.015	1.460	4.488	0.034
Histrionic PD	−0.185	0.089	0.831	0.698	0.989	4.366	0.037
**Sexual abuse as the dependent variable**							
Antisocial PD	0.187	0.105	1.205	0.982	1.479	3.178	0.075
Histrionic PD	−0.193	0.101	0.825	0.676	1.005	3.645	0.056
Narcissistic PD	0.176	0.104	1.193	0.973	1.462	2.885	0.089
**Physical neglect as the dependent variable**							
Negative symptoms	0.053	0.025	1.054	1.005	1.107	4.609	0.032
**Emotional neglect as the dependent variable**							
Dependent PD	−0.160	0.073	0.852	0.739	0.982	4.885	0.027

Beta is the regression coefficient. S.E. is the standard error. 95% CI is the estimated 95% confidence interval for the corresponding parameter. β is the standardized regression coefficient. PD, personality disorder.

## Discussion

### Key findings

Consistent with previous evidence, our findings show that ACEs are very common in patients with schizophrenia. The vast majority of participants reported the neglect type of ACE, especially physical neglect, and over two-thirds of patients with schizophrenia reported experiencing physical neglect during childhood. Further, we found that ACEs were associated with psychotic symptoms, mainly negative symptoms, and personality pathology. Regarding specific ACE, the abuse type of ACEs was likely to be associated with personality pathology, such as antisocial PD traits, whereas the neglect type of ACEs was likely to be associated with negative symptoms. This study holds significant value in advancing our understanding of the relationship between childhood trauma, personality disorders, and schizophrenia. Its comprehensive assessment approach and large sample size provide valuable insights into the complex interplay of these variables. The findings highlight the importance of addressing ACEs and personality traits in the clinical management of individuals with schizophrenia.

### ACEs frequency

This study contributes to the literature on the frequency and potential associations of schizophrenia among individuals who have experienced ACEs. Our findings indicated a higher occurrence of ACEs, with neglect being a significant factor reported by patients with schizophrenia. These results align with those of previous research suggesting a link between childhood trauma and the development of psychotic disorders (25–27). The higher prevalence of childhood neglect in patients with schizophrenia raises important questions regarding their potential causal relationship (28). One possible explanation is that early life neglect disrupts the development of secure attachment bonds and leads to impaired socioemotional functioning, which may contribute to the development of schizophrenia (5). Neglect-type ACEs may also affect neurobiological and neurodevelopmental processes, resulting in alterations in the brain structure and function associated with the disorder (29–31). Neglect refers to the absence of proper care, emotional support, and attention to a child’s basic needs. This chronic deprivation of essential nurturing experiences during critical periods of brain development can lead to lasting alterations in brain structure and function. During early childhood, the brain undergoes rapid growth and refinement of neural connections, known as neuroplasticity. Neglect disrupts this delicate process, hindering the formation of healthy neural circuits and synaptic connections. This can result in abnormal brain development, particularly in regions crucial for emotional regulation, cognitive processing, and social interaction, which are relevant to the pathology of schizophrenia. Understanding the relationship between ACEs and schizophrenia has important implications for both clinical practice and public health. Early identification and intervention in individuals who have experienced ACEs can potentially mitigate the risk or severity of developing schizophrenia (32). Moreover, promoting awareness regarding the impact of childhood neglect on mental health may help improve support systems for patients with schizophrenia.

### Association with negative symptoms

Negative symptoms, which encompass deficits in emotional, cognitive, and social functioning, are the core features of schizophrenia (33). Consistent with previous studies (34, 35), the present study suggests a possible link between negative symptoms and ACEs, particularly neglect. There are several potential reasons for the observed relationship between negative symptoms of schizophrenia and ACEs. First, ACEs can have a profound impact on psychological well-being, leading to the development of maladaptive coping mechanisms and emotional dysregulation (36). These psychological factors may contribute to the emergence and persistence of negative symptoms in patients with schizophrenia (37). Additionally, ACEs can result in chronic stress and an altered stress response system (38), which have been associated with the negative symptoms of schizophrenia. Furthermore, social factors influenced by ACEs such as disrupted attachment patterns, dysfunctional family dynamics, and social isolation may contribute to the development and maintenance of negative symptoms. ACEs can impair social and interpersonal skills, leading to difficulties in forming and maintaining relationships as well as reduced motivation to engage in social activities (29).

### Association with personality pathology

Our study further explored the association between ACEs and personality pathology in patients with schizophrenia, with a particular focus on the potential mediating roles of PD traits. The findings of this study provide valuable insights into the complex relationships between childhood abuse, personality pathology, and schizophrenia. Consistent with previous studies (39, 40), our results revealed a significant positive correlation between ACEs and personality pathology in patients with schizophrenia. This finding supports previous research that demonstrated the detrimental impact of childhood abuse on the development of personality pathologies. ACEs may contribute to the formation of maladaptive personality traits such as impulsivity, emotional dysregulation, and interpersonal difficulties, which are commonly observed in patients with schizophrenia. Furthermore, our results suggest that PD traits may partially mediate this association. Specifically, we found that certain personality traits, such as antisocial PD traits, partially accounted for the relationship between childhood abuse and schizophrenia.

### Implications for psychological treatment

The results of this study have important implications for psychological treatment interventions in individuals with schizophrenia who have experienced ACEs, particularly neglect-type ACEs. Firstly, the findings highlight the importance of trauma-informed care in the treatment of schizophrenia. It is crucial for mental health professionals to be aware of and sensitive to the presence of ACEs and their potential influence on symptomatology and personality pathology. Secondly, addressing the impact of neglect-type ACEs on social and interpersonal functioning is essential. The deficits in social cognition and attachment bonds resulting from neglect can significantly affect relationships and social interactions in individuals with schizophrenia. Therefore, interventions that focus on improving social skills, enhancing interpersonal relationships, and fostering a sense of belonging and connection can be beneficial. Additionally, the findings emphasize the need for comprehensive treatment approaches that integrate both symptom management and trauma resolution.

### Limitations

Although this study provides valuable insights into the research topic, it is important to consider the following limitations when interpreting and applying the findings. First, the use of a cross-sectional design prevented the establishment of causal relationships between ACEs and schizophrenia. It provides only a snapshot of the data at a specific time point, making it difficult to determine the directionality of the observed associations. Second, the reliance on subjective measures through scales and questionnaires introduced the potential for bias in participant responses. Subjective evaluations may be influenced by personal experiences, beliefs, or social desirability, which can affect the validity and reliability of results. Third, the study may have been susceptible to recall bias as participants were required to remember past events and experiences. Recall bias can arise because of memory inaccuracies or selective memory, leading to errors or incomplete information that may affect the validity and generalizability of the findings. Fourth, the findings may lack generalizability because of the use of a single-center sample. Fifth, it is important to acknowledge that our study did not specifically evaluate the utilization and effects of potentially protective factors, such as psychotherapy and counseling interventions, which individuals frequently pursue without specialist prescription to prevent or alleviate symptoms during the onset phase. Although these interventions are commonly adopted by individuals recognizing early signs of mental health concerns, our study did not emphasize their assessment. Future research endeavors could contemplate the inclusion of measurements targeting the utilization and potential advantages of these interventions. Finally, the lack of a healthy control group is a limitation of this study. Future research with different study designs, objective measures, larger samples, and multi-center collaborations would be beneficial to address these limitations and enhance the overall understanding of the topic.

## Conclusion

Several ACEs, particularly physical neglect, have been reported in patients with schizophrenia. Each ACE type was found to be associated with different symptoms and personality pathologies. Specifically, abuse was closely related to personality pathology, whereas neglect was associated with negative symptoms. Future research should further explore the complex relationships among ACEs, psychotic symptoms, and personality pathology. Longitudinal studies can provide valuable insight into the trajectories and progression of these effects over time. Such knowledge could ultimately inform the development of more effective interventions and treatment approaches for patients with schizophrenia and history of ACEs.

## Data availability statement

The original contributions presented in the study are included in the article/supplementary material, further inquiries can be directed to the corresponding author.

## Ethics statement

This study was reviewed and approved by the ethics committees of the Shanghai Pudong New Area Mental Health Center, Tongji University School of Medicine. The patients/participants provided their written informed consent to participate in this study.

## Author contributions

LZ, WH, and WL conceived and designed the experiments. LZ, WH, NZ, MZ, and MT performed the experiments. LZ, WH, and WL analyzed and discussed the data. LZ and WH were major contributors in writing the manuscript. All authors contributed to the article and approved the submitted version.

## Funding

This work was supported by grants from Science and Technology Development Fund of Shanghai Pudong New Area (PKJ2022-Y77), Pudong New Area Health Committee Disciplinary Leader Training Program (No. PWRd2021-06), Shanghai Municipal Health Industry Clinical Research Project (No. 20204y0194), and Key Clinical Discipline Project of Pudong (No. PWZzk2022-19; No. PWYgy2018-10; PDJWM-202104).

## Conflict of interest

The authors declare that the research was conducted in the absence of any commercial or financial relationships that could be construed as a potential conflict of interest.

## Publisher’s note

All claims expressed in this article are solely those of the authors and do not necessarily represent those of their affiliated organizations, or those of the publisher, the editors and the reviewers. Any product that may be evaluated in this article, or claim that may be made by its manufacturer, is not guaranteed or endorsed by the publisher.
